# Thrombospondin-1 Modulates Actin Filament Remodeling and Cell Motility in Mouse Mammary Tumor cells in Vitro

**DOI:** 10.15190/d.2014.23

**Published:** 2014-10-08

**Authors:** Dorothy Ndishabandi, Cameron Duquette, Ghita El-Moatassim Billah, Millys Reyes, Mark Duquette, Jack Lawler, Shideh Kazerounian

**Affiliations:** Department of Pathology, Division of Experimental Pathology, Beth Israel Deaconess Medical Center and Harvard Medical School, Boston, MA; Department of Pediatrics, Division of Genetics, Boston Children’s Hospital and Harvard Medical School Boston, MA

**Keywords:** Thrombospondin-1, integrin α3β1, metastasis, actin filaments, breast cancer

## Abstract

It is well established that the secretion of thrombospondin-1 (TSP-1) by activated stromal cells and its accumulation in the tumor microenvironment during dysplasia inhibits primary tumor growth through inhibition of angiogenesis. This inhibitory function of TSP-1 is actuated either by inhibiting MMP9 activation and the release of VEGF from extracellular matrix or by an interaction with CD36 on the surface of endothelial cells resulting in an increase in apoptosis. In contrast, several published articles have also shown that as tumor cells become more invasive and enter the early stage of carcinoma, they up-regulate TSP-1 expression, which may promote invasion and migration. In our in vivo studies using the polyoma middle T antigen (PyT) transgenic mouse model of breast cancer, we observed that the absence of TSP-1 significantly increased the growth of primary tumors, but delayed metastasis to the lungs. In this study, we propose a mechanism for the promigratory function of TSP-1 in mouse mammary tumor cells in vitro. We demonstrate the correlations between expression of TSP-1 and its receptor integrin α3β1, which is considered a promigratory protein in cancer cells. In addition we propose that binding of TSP-1 to integrin α3β1 is important for mediating actin filament polymerization and therefore, cell motility. These findings can help explain the dual functionality of TSP-1 in cancer progression.

## INTRODUCTION

Thrombospondin-1 (TSP-1) is a 450 kDa trimeric multi-domain extracellular calcium-binding glycoprotein that is expressed by most cell types including fibroblasts, platelets, endothelial cells, monocytes, and epithelial cells. TSP-1 binds to a variety of cell surface receptors and extracellular matrix proteins in a cell and concentration-dependent manner and is involved in a variety of biological processes including platelet aggregation, inflammation, and the regulation of angiogenesis during wound repair and tumor growth^1-32^. Moreover, TSP-1 acts as a scaffold to bring receptors and growth factors into close proximity to ultimately orchestrate complex signaling cascades^23,32^. In addition to full-length TSP-1, proteolytic fragments of TSP-1 are also able to interact with numerous receptors and ligands to mediate cellular events which differ from the intact TSP-1 protein^21,29^. In endothelial cells, TSP-1 can bind to CD36 and CD47 through its type-1 repeats and carboxyl-terminal domain to inhibit cell proliferation and promote anti-angiogenic events^8,16^. The amino-terminal heparin binding domain of TSP-1 also interacts with a variety of cell surface proteins, including heparin sulfate proteoglycan, calreticulin, and integrins such as a3β1 to modulate cell adhesion and motility in a cell specific manner^9,10,12,22,29^. In the tumor microenvironment, secretion of TSP-1 by activated stromal cells including fibroblasts inhibits primary tumor growth and invasiveness by inhibiting MMP9 and therefore the release of VEGF from extracellular matrix or by binding to CD36 on the surface of endothelial cells and promoting apoptosis^3,18,30^. However, recently published studies have suggested that as some tumor cells become more invasive and enter the early carcinoma stage, they up-regulate TSP-1 expression, which may play a role in promoting their migration^4,18,24^. Nucera, et al, also reported similar findings in thyroid cancer cells with the B-Raf mutation^28^. They showed that these cells up-regulated TSP-1 expression and displayed a higher level of adhesion, migration, and invasion *in vitro* compared to cancer cells with B-RafV600E knockdown^28^. Moreover, knockdown of TSP-1 in 8505c thyroid cancer cells also resulted in decreased adhesion, migration and invasion^28^. They also demonstrated that a reduction in TSP-1 expression in anaplastic thyroid cancer cells was accompanied by a decrease in the expression levels of integrin α3, α6, and β1 and a change in cell morphology^11^. We also reported opposing functions of TSP-1 in our *in vivo* studies using the polyoma middle T antigen (PyT) transgenic mice model of breast cancer, which mimics the progression of this disease in humans^33-37^. In our studies, primary tumors in TSP-1-null mice grew faster than tumors in wild-type mice. In contrast, at 90 days, the number of metastatic lesions in the lungs was higher in the wild-type animals than in TSP-1-null PyT mice. In this study, we hypothesized that endogenous expression of TSP-1 and its receptor integrin a3β1 may enhance migration of breast cancer cells. Integrin a3β1 is known to mediate actin filament polymerization and cell motility. To address this hypothesis, we performed *in vitro* studies using mammary tumor cells isolated from wild-type and TSP-1-null PyT mice at 90 days of age. We quantified the expression level of integrin receptors of TSP-1 including a3β1 in wild-type and TSP-1-null tumor cells. We also compared the morphology and the organization of actin filaments in wild-type and TSP-1-null tumor cells. Our results suggest a correlation between TSP-1 and integrin a3β1 expression both at the protein and transcription levels. However, this correlation was not detected in real-time PCR of mRNA isolated from tumor tissues suggesting the possibility that cells in culture may display changes in gene expression over time. We consider this difference an important consideration when comparing *in vitro *and* in vivo* studies, identifying new targets, and developing new therapies.

## MATERIALS AND METHODS**

### Antibodies and Reagents

Anti-integrin a3 subunit (sc-6587) and aV subunit (sc-10719) were obtained from Santa Cruz Biotechnology Inc (Santa Cruz, CA)^19,21^. The rabbit polyclonal anti-serum to the integrin b1 subunit was a gift from Dr. Richard Hynes (Howard Hughes Medical Institute, Massachusetts Institute of Technology, Cambridge, MA)^19^. HRP-conjugated goat anti-rabbit IgG (7076) and anti-mouse IgG (7074) were obtained from Cell Signaling Technology (Danvers, MA). The anti-a smooth muscle actin (clone A4) was obtained from Sigma Aldrich (Milwaukee, WI)^19^. Antibody to fibroblast activation protein (FAP) was a generous gift from Dr. Jonathan D. Chang (Fox Chase Medical Center, Philadelphia, PA)^22^. The TSP-1 rabbit polyclonal antibody (R1) was produced in our lab by immunizing rabbits with purified full-length platelet TSP-1^23^. The TSP-1 mouse monoclonal antibody (MAI) has been described previously^20^. **

### Cell Culture

Human breast cancer cell lines (MDA-MB-231, MCF-7) and normal mammary epithelial cells (MCF-10A) were generously provided by Dr. Joan Brugge (Department of Cell Biology, Harvard Medical School, Boston, MA). Sum-159pt and MDA-MB-468 were a generous gift from Dr. Alex Toker (Beth Israel Deaconess Medical Center, Boston MA). CommA-1D normal mouse epithelial cells were a generous gift from Dr. Bassem R. Haddad (Georgetown University, Washington, D.C.) Mouse mammary tumor cells were isolated from wild-type or TSP-1-null PyT mice at 90 days of age, as described previously^37^. All cells were maintained in (1:1) DMEM/Ham’s F12 media (10-090-CV) from Mediatech (Manasar, VA) supplemented with EGF (20ng/ml), hydrocortisone (0.5mg/ml), insulin (10mg/ml), and antibiotics. Sum-159pt cells were grown in Ham’s F12 medium containing 5mg/ml insulin, 1 mg/ml hydrocortisone, and 5% fetal bovine serum.**

### Immunoassay Analysis of Human Breast Cancer Cells and Mammary Tumor Cells Isolated from PyT Mice

The cell extracts were prepared with 1% Triton X-100 lysis buffer containing (20 mM HEPES PH 7.5, 150 mM NaCl, 5 mM EDTA, 1mM Na_3_VO_4_, 20 mM NaF, 1mM EGTA, and protease inhibitors (Pierce)) for 20 minutes at 4˚C, The lysates were centrifuged at 12,000 rpm for 20 minutes at 4˚C. The cell lysates were either used immediately for immunoassay experiments or stored at -80˚C. To determine the expression level of proteins in each cell type, 15 mg of total cell extract was subjected to SDS-PAGE. Proteins were transferred to a nitrocellulose membrane (Bio-Rad Laboratories, Hercules, CA), and the membrane was blocked in 5% non-fat dry milk in TBST (10 mM Tris-HCl (PH 7.4), 150 mM NaCl, with either 0.1% or 0.05% Tween-20) for 1 hour at room temperature. The primary antibodies were prepared in blocking solution at a 1:500 dilution and incubated overnight except for MAI (1:1000), R1 (1:3000), and integrin b1 (1:3000), which were incubated at room temperature for 1 hour with mixing. After 5 washes for 5 minutes each in TBST, the HRP-conjugated secondary antibody was added, and the membrane was incubated for 1 hour at room temperature. The membrane was washed 5 times for 5 minutes each in TBST, and the bands were visualized using ECL detection (34080) from Thermo Scientific (Rockford, IL) or (WBKLS0100) from Millipore (Billerica, MA).**

### Cell Migration Assay

The migration assay was performed using Boyden chambers with polycarbonate filters with a 8μm pore size (Corning Inc, Corning, NY). Human breast cancer cells (MDA-231 and MCF-7), mammary tumor cells from wild-type or TSP-1-null PyT mice at 90 days of age, human normal mammary epithelial cells (MCF-10A), and mouse normal mammary epithelial cells (COMMA-1D) were serum-starved overnight in growth media with 0.1% BSA. Each side of the membrane was coated with 50 μg /ml of collagen I in PBS for 1 hour at room temperature. Cells were washed, counted, and suspended in PBS with 0.1% BSA. 400 μl of a 20,000 cells/ml suspension was added to the upper chamber in duplicate and incubated at 37°C for 90 minutes. 400 ml of 3T3 conditioned medium (chemoattractant) or 1% BSA in PBS (negative control) was added to the lower chamber, and the incubation continued for another 5 hours. Non-migrating cells were removed from the upper membrane with Q-tips, and the membrane was fixed in methanol for 2 minutes and Geisma stain (Richard-Alan Scientific, Kalamazoo, MI) for 30 minutes. Four fields were counted at 20X magnification, and the average number of migrated cells was calculated. The error bars indicate the standard error mean. P values are based on the unpaired student’s t-test with 2-tail distribution.**

### Invasion Assay

Mammary tumor cells from wild-type and TSP-1-null PyT mice at 90 days of age and normal mouse mammary epithelial cells (CommA-1D) were serum-starved overnight as described previously. The invasion assay was performed using an invasion kit from BD Biosciences (BD Biocoat 354480). Briefly, the transwells were rehydrated with 500 ml of serum free culture medium with 0.1% BSA for 2 hours at 37°C, and 200 mls of a 1X10^4 ^cells/ml of culture medium with 0.1% BSA and without serum was plated on the transwells. 750 mls of 3T3 conditioned medium (chemo-attractant) or 1% BSA in PBS (negative control) were used in lower wells. Cells were allowed to invade for 6 hours at 37°C. Cells were fixed and stained as described for migration assays. Four fields in the lower filter were counted at 20X magnification, and the average invaded cells were calculated. The error bars indicate the standard error mean. P values are based on the unpaired student’s t-test with 2-tail distribution.**

### Indirect immunofluorescence

To perform this experiment, coverslips were coated with 50 mg/ml of full-length TSP-1 or collagen type I for 2 hours at room temperature. Mammary tumor cells from wild-type or TSP-1- null PyT mice at 90 days of age were plated on coverslips and incubated overnight at 37°C. The next day, cells were washed in PBS and fixed in 4.7% paraformaldehyde in PBS, and permeabilized in 0.05% Triton X-100 in PBS. After blocking (20 mM Tris (PH 7.5), 150 mM NaCl, 1% BSA) for an hour at room temperature, cells were stained with 1:500 dilution of isothiocynate-labeled phalloidin (P5282) from Sigma Aldrich (Milwaukee, WI) for 30 minutes at room temperature. Coverslips were mounted with VECTAshield mounting medium with DAPI (H-1200) from Vector Labs Inc (Burlingame, CA). The slides were examined using a ZEISS fluorescence microscope Imager.A1 equipped with a ZEISS camera and analyzed using IVision (BioVision Technologies, Inc) software. For morphology studies, cells were visualized under bright light at 20X on a Nikon Eclipse 300 epifluorescence inverted microscope connected to a Nikon camera.

### Real-time PCR

RNA was isolated from tumor cells from wild-type and TSP-1-null PyT mice at 90 days of age using a RNeasy Mini kit (74104) from Qiagen, Inc (Valencia, CA) according to manufacturer’s protocol. cDNA was prepared using reverse transcriptase III (Invitrogen) according to manufacturer’s protocol. The mRNA copy number per cell was determined using MGTP, a form of quantitative real-time PCR, and was calculated by normalization to 18S rRNA abundance assuming that each cell, on average, expresses about 10^6^ copies. The mean and standard error of the mean (mean + SEM) were calculated from experiments that were done in duplicates.

## RESULTS

### Isolation of Mouse Mammary Tumor Cells

**Figure 1:**
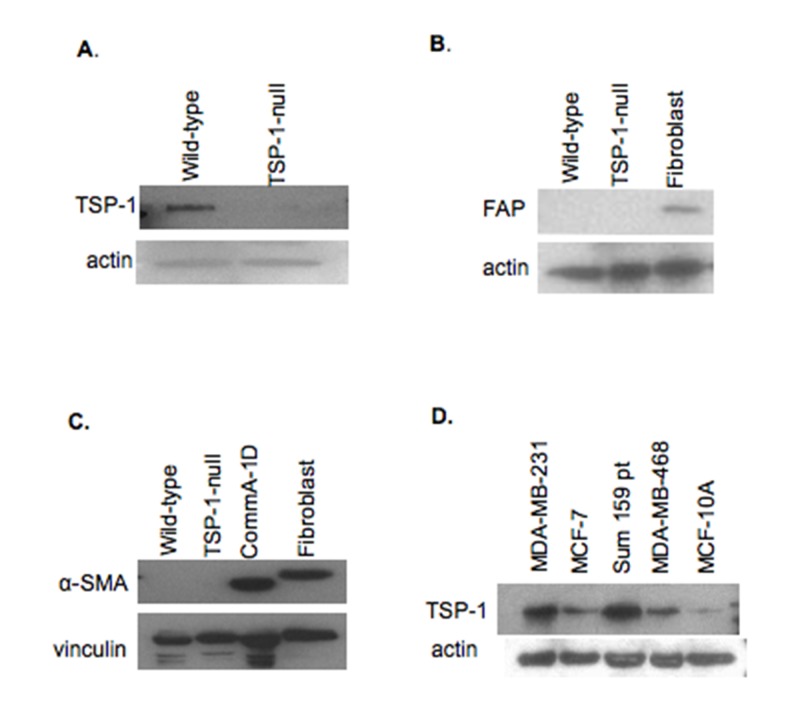
Isolation of Mouse Mammary Tumor Cells and Detection of TSP-1 Expression in Breast Cancer Cells Mammary tumor cells were isolated from PyT mice, as described by Yee et al^37^. The cell extracts were subjected to immunoblot analysis. (A) Detection of TSP-1 in tumor cells isolated from PyT mice at 90 days of age. (B and C) Absence of fibroblast markers, fibroblast activated proteins (FAP) and α-smooth muscle actin (α-SMA) in the population of isolated wild-type and TSP-1-null tumor cells. (D) Comparison of TSP-1 expression level among various human breast cancer cell lines.

To investigate the role of TSP-1 expression in mediating cancer cell migration, we isolated tumor epithelial cells from mammary tumors of wild-type and TSP-1-null PyT mice at 90 days of age (corresponding to late carcinoma in humans)^37^. While comparing tumor metastasis to the lungs between wild-type and TSP-1-null PyT mice in our* in vivo* studies^37^, we observed a higher number of lung lesions in wild-type mice at 90 days of age with a significant difference being detected as early as 45 days of age. Therefore, It was reasonable to utilize mammary epithelial cells from 90-day tumors to better understand the mechanism by which TSP-1 regulates tumor cell migration. To ensure our tumor epithelial cell isolates were free of fibroblasts, we tested our cells for the presence of a-smooth muscle actin and fibroblast activated protein (FAP), markers for tumor-associated fibroblasts^19,37^. Tumor cells were lysed in 1% Triton X-100 (described in Materials and Methods), and subjected to immunoblot analysis using antibodies specific to TSP-1, a-smooth muscle actin, and fibroblast activated proteins (FAP). The results showed absence of TSP-1 in null cells, as expected (**Figure 1** [A]). High levels of smooth muscle actin and fibroblast activated protein expression were detected in tumor-associated fibroblasts, which were also isolated in our laboratory, both proteins were absent from isolated tumor cells (**Figure 1** [B, C]). In this experiment, CommA-1D, normal mouse mammary epithelial cells^7^, was used as a control.

### Detection of Endogenous Expression of TSP-1 in Breast Cancer Cells

Next, we examined the correlation between the endogenous expression level of TSP-1 and the rate of migration in a variety of human breast cancer cells. Breast cancer cells were lysed in 1% Triton X-100 lyisis buffer, and equal amounts of total lysate were subjected to immunoblotting using antibodies against TSP-1. The results showed that MDA-MB-231 and Sum 159pt, estrogen receptor negative and highly invasive breast cancer cells, expressed a high level of TSP-1 (**Figure 1** [D]). In contrast, TSP-1 was moderately expressed in the estrogen receptor positive and less aggressive MCF-7 cells and non-migratory MDA-MB-468 breast cancer cells and was barely detectable in MCF-10A normal mammary epithelial cells (**Figure 1** [D]). These findings were in agreement with similar *in vitro* studies published by other investigators suggesting that an increase in the level of TSP-1 expression in breast cancer cells associated with a higher migration rate^1,2,34^.

### Invasion and Migration of Mouse Mammary Tumor Cells and Human Breast Cancer Cells

**Figure 2:**
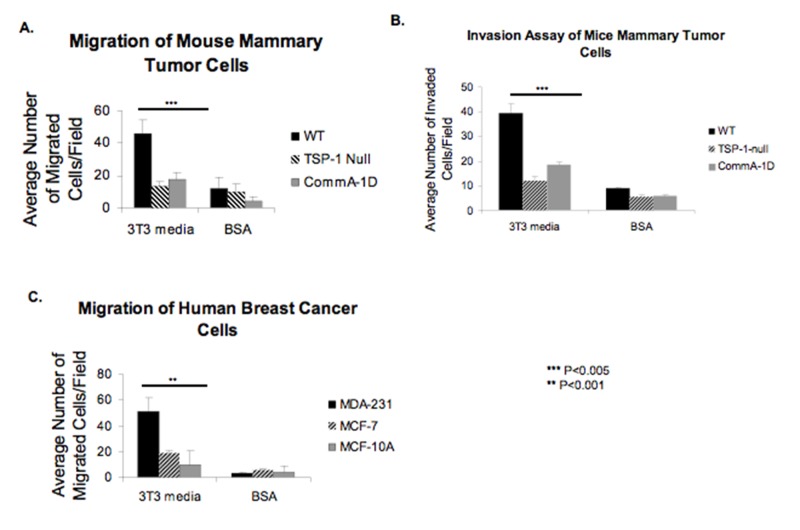
In Vitro Migration and Invasion of Mammary Tumor Cells Isolated from Wild-type and TSP-1-null PyT Mice and Human Breast Cancer Cells. (A and C) Mammary tumor cells from wild-type or TSP-1-null PyT mice at 90 days of age, mouse normal mammary epithelial cells (CommA-1D), human breast cancer cells (MDA-231 and MCF-7), and normal mammary epithelial cells (MCF-10A) were serum-starved overnight in DMEM culture medium and Migration assay was performed by Boyden’s chamber. Four fields in the lower filter were counted at 20X magnification, and the average migrated cells were calculated. The results show that the wild-type tumor cells migrate with a significantly higher rate compared with the null tumor cells and normal epithelial cells. Similar results are observed for human breast cancer cells. MDA-MB-231 cells have a significantly higher migration rate toward 3T3 conditioned media compared with MCF-7 and MCF-10A cells. (B) Mammary tumor cells from wild-type and TSP-1-null PyT mice at 90 days of age and normal mouse mammary epithelial cells (CommA-1D) were serum starved overnight as described previously. The invasion assay was performed using invasion kit from BD Biosciences (BD Biocoat 354480). Four fields in the lower filter were counted at 20X magnification, and the average invaded cells were calculated. As it is observed for the migration assay, the wild-type tumor cells have a higher invasion rate compared with the null tumor cells and normal epithelial cells. The error bars in both experiments indicate the standard error mean. P values are based on the unpaired student’s t-test with 2-tail distribution.

The possibility that tumor epithelial cell TSP-1 may be important for the pro-invasiveness and pro-migratory characteristics of mammary tumor cells was further examined through migration and invasion studies. Mammary tumor cells from wild-type and TSP-1-null tumors at 90 days of age as well as mouse normal mammary epithelial cells (CommA-1D) were serum-starved overnight in DMEM culture medium with 0.1% BSA. *In vitro* invasion and migration assays were performed as described in Materials and Methods. Compared with CommA-1D cells and TSP-null tumor cells, wild-type cells displayed a significantly higher migration and invasion rate (**Figure 2** [A, B]). In contrast, both TSP-1-null and normal epithelial cells demonstrated very similar migration and invasion rates (**Figure 2** [A, B]). In parallel studies, we also compared the migration rate of MDA-MB-231 and MCF-7 breast cancer cells. In these studies MCF-10A cells were used as a control. The results showed a significantly higher migration rate for MDA-MB-231 cells, with the highest expression of endogenous TSP-1 (**Figure 2** [C]). However, there was no significant difference between the migration rate of MCF-7 cells and MCF-10A cells (**Figure 2** [C]). Together, these finding further support the possibility that TSP-1 secreted by tumor cells and not by stromal cells such as fibroblasts, was responsible for their invasion and migration.

### Morphological Analysis of Mouse Mammary Tumor cells Isolated from Wild-type and TSP-1-null PyT Mice

**Figure 3:**
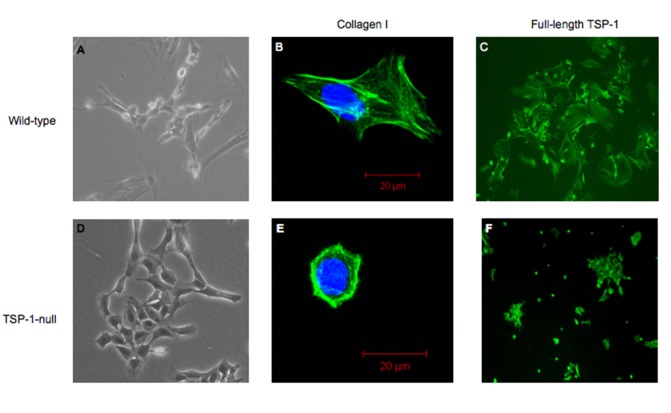
Actin Microfilament Organization in Wild-type and TSP-1-null Mammary Tumor Cells Mammary tumor cells isolated from 90-day wild-type or TSP-1-null PyT mice were cultured in complete media (as described in Materials and Methods). They were either examined directly under microscope or plated on coverslips coated with 50 μg/ml of full length TSP-1 or collagen type I and incubated overnight at 37ºC and stained with isothiocynate-labeled phalloidin (as described in Materials and Methods). (A and D) Cells were visualized under bright light at 20X on a Nikon Eclipse 300 epifluorescence inverted microscope connected to a Nikon camera. The wild-type tumor epithelial cells are attached and flattened, while the TSP-1-null cells remain rounded. (B and E) Tumor cells were seeded on collagen I-coated plates, and the actin filament polymerization was examined under a ZEISS fluorescence microscope imager.A1 equipped ZEISS camera and analyzed using IVision (BioVision Technologies, Inc) software. The results suggest that in wild-type tumor cells, actin filaments have retained their structure and are organized in filaments, while in the null tumor cells, the filamentous structure is not present. (C and F) Tumor cells were allowed to attach to full-length TSP-1, and actin filament were examined at 20X on a Nikon Eclipse 300 epifluorescence inverted microscope connected to a Nikon camera. The results show that both wild-type and null tumor cells attach to the TSP-1, but there is no change in their morphology. The wild-type cells are flattened while the majority of null tumor cells are rounded.

We also examined potential morphological differences between wild-type and TSP-1-null mouse mammary tumor cells, which may also play a role in their migration behavior. Cells were grown in 6-cm dishes as a monolayer in conditioned media supplemented with necessary growth factors and visualized under light microscope (**Figure 3** [A, D]). The majority of null tumor cells attached but remained rounded, while wild-type tumor cells underwent spreading and became flattened (**Figure 3** [A, D]). Similar results were observed when cells were allowed to attach to collagen I-coated coverslips and stained with phalloidin^13,31^ to visualize F-actin under confocal microscope. Whereas wild-type cells exhibited organized actin filaments and well-spread morphology, the majority of TSP-1-null tumor cells remained rounded (**Figure 3** [B, E]). To determine if the cells treated with full-length TSP-1 would rescue the organization of actin filaments, cells were allowed to attach to coverslips coated with 50 μg/ml of full-length platelet TSP-1^36^ over night and stained with phalloidin (**Figure 3** [C, F]). However, we did not observe any changes in the organization of stress fibers. In parallel studies, we also compared the morphology of MDA-MB-231 and MCF-7, and observed similar differences (data not shown). The noticeable changes in morphology and actin filament organization between wild-type and TSP-1-null tumor cells led us to investigate the signaling pathways that could be initiated by TSP-1 to modulate the cytoskeletal structures of tumor cells.

### Differential Expression of Integrins in Wild-type and TSP-1-null Mammary Tumor Cells

**Figure 4:**
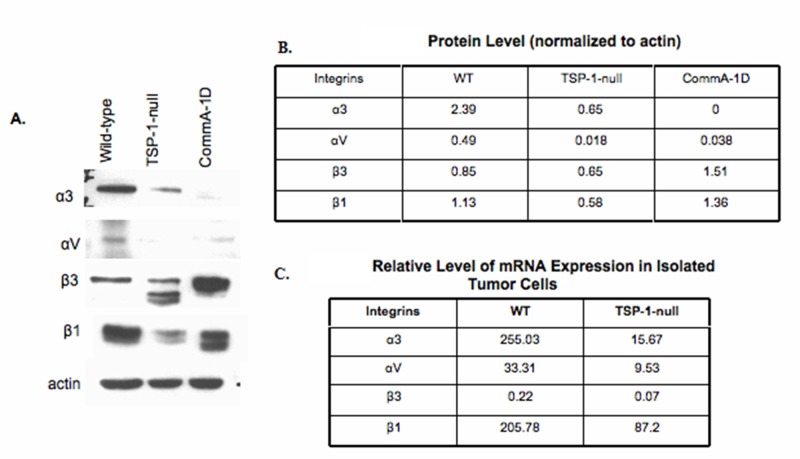
Comparison of the Expression of Integrin Subunits at Protein and mRNA levels in Mammary Tumor Cells Isolated from Wild-type and TSP-1-null 90-day PyT mice (A) Mouse mammary tumor cells were lysed in 1% Triton X-100 lysis buffer (as described previously). To determine the expression level of proteins in each cell type, 15 µg of total protein extract was subjected to immunoblot analysis, and the level of expression was normalized to actin (B). The results suggest a higher expression levels of integrins α3, αv, and β1 subunits in the wild-type tumor cells compared with the TSP-1-null tumor cells, while both cell type express similar levels of integrin β3 subunit. RNA was extracted from the same tumor cells, and cDNA was prepared using reverse transcriptase III (Invitrogen). qPCR was performed using primers against different integrin subunits (C). The mRNA copy numbers per cell was calculated by normalization to 18S rRNA abundance assuming that each cell, on average, expresses about 106 copies. The mean and standard error of the mean (mean + SEM) were calculated from experiments that were done in duplicates. The results show that integrins α3, αv, and β1 subunits also have higher levels of transcription expression in the wild-type tumor cells compared with the TSP-1-null tumor cells. Again, both cell type express similar levels of integrin β3 subunit.

To address a possible mechanism for the morphological differences between the wild-type and TSP-1-null tumor cells, we compared the expression levels of various integrin subunits including a3, β1, and β3, which are known to bind to TSP-1^22,25^. Mammary tumor cells were lysed in 1% Triton X-100 lysis buffer and an equal amount of total lysate was subjected to immunoblotting using antibodies against various integrin subunits. In parallel studies, RNA was isolated from the same cells and the relative expression of integrin mRNA was analyzed for each cell type. The most significant changes were detected for integrin α3 and β1 both by immunoblot analysis and relative quantification of mRNA (**Figure 4** [A-C]). We should also note that even though CommA-1D cells expressed a high level of integrin β1 subunit, the level of integrin α3 was barely detectable. We believe that these integrin subunits have to be expressed at similar levels to form a functional integrin receptor. This could address the question as to why CommA-1D cells have a similar migration rate as TSP-1-null tumor cells. Taken together, these results suggest the possibility that the cellular events, which are modulated by TSP-1, are cell-type specific and are highly regulated by the expression levels of one or more of the TSP-1 receptors in that particular cell type. Integrin a3b1 is well known for its pro-migratory function in a variety of cancer cell types, so it is worth mentioning that it may also be responsible for mediating the pro-migratory function of TSP-1 in breast cancer cells.

## DISCUSSION

Although significant progress has been made in detecting and treating primary tumors, the ability to predict the metastatic behavior of tumors remains a major clinical challenge. Metastasis is a multistep event that allows tumor cells to invade through extracellular matrix and migrate to distant organs. Therefore, the ability of tumors to grow and progress involves the crosstalk between tumor cells and activated stromal cells and is reguated by a balance between pro-angiogenic (VEGF-A) and anti-angiogneic (TSP-1) factors.

During primary tumor formation, the secretion of VEGF-A by tumor cells induces endothelial cell proliferation and migration and initiates angiogenesis and blood vessel sprouting to increase tumor cell growth. In contrast, secretion of TSP-1 by activated stromal cells including fibroblasts inhibits tumor growth by binding to CD36 on the surface of endothelial cells and promoting apoptosis^3,18,30^.

Interestingly, several earlier reports suggested that TSP-1 was also able to promote tumor cell migration in a variety of cancers such as melanoma and prostate cancer^14,17^. Both TSP-1 and CD36 are also detected in normal, hyperplasic, and neoplastic human breast tissue^6,34,35^. Moreover, the co-distribution of TSP-1 and CD36 in a subpopulation of invasive lobular carcinoma correlates with the capability of these cells to invade through surrounding stroma^34,35^. The possible pro-migratory function of TSP-1 has further been demonstrated by our *in vivo* studies using the polyoma middle-T antigen (PyT) transgenic mice model of breast cancer^37^. We reported that tumors in TSP-1-null mice grew faster than the wild-type mice. In contrast, at 90 days of age, the number of metastatic lesions in the lungs was higher in the wild-type than TSP-1-null mice. This difference was also detectable when comparing wild-type and TSP-1-null PyT mice at 45 days of age^37^.

In this study, we have utilized tumor cells isolated from wild-type and TSP-1-null PyT mice to address the molecular mechanism by which TSP-1 promoted breast cancer cell migration. First, we showed that the more migratory breast cancer cells also expressed higher levels of TSP-1. Moreover, lack of endogenous expression of TSP-1 also affected the organization of actin filaments and spreading of cells in culture. TSP-1 is a very complex protein with several binding sites for a variety of cell surface receptors. Therefore, we hypothesized that the opposing functions of TSP-1, as an inhibitor of tumor cell proliferation and tumor growth and a mediator of tumor cell invasion and migration, could be related to differential expression of its receptors in various cell types. For example, the amino-terminal domain of TSP-1 binds to integrin a3b1. The role of this integrin in promoting cell invasion and motility by up-regulating MMP2 and MMP9 as well as cytoskeletal organization in a PI3K-dependent manner has been reported for a variety of cancers^5,15,27,38^.Therefore, in this study, we also examined the expression levels of various integrin subunits, which are known to bind to TSP-1, both at a protein and transcription levels in wild-type and TSP-1-null mammary tumor cells. Interestingly, our results suggested a higher expression of integina3b1 in wild-type compared with TSP1-null mammary tumor cells. These results were in agreement with findings by other investigators suggesting that suppression of integrin a3b1 reduced the migration of breast cancer cells^26,27^. However, we should mention that because the observed change in the expression of integrin a3 and β1 subunits was detected only in our *in vitro *studies, further studies are required to investigate these findings *in vivo *or possibly by *in vitro *recapitulating of the tumor microenvironment.

Overall, our findings in this study present new insights into the signaling pathways that mediate cell invasion and motility. We suggest that TSP-1 can exert its pro-migratory function by bringing various receptors into close proximity at the surface of tumor cells to initiate signaling pathways which modulate gene expression and actin filament formation and ultimately affecting cell invasion and migration. Our results also suggest a new approach for identifying which domain of TSP-1 to target during cancer progression for developing targeted therapeutics.

## KEY POINTS

**◊ Thrombospondin-1 facilitates breast cancer cell migration in vitro**.
